# Single-cell RNA sequencing in melanoma: what have we learned so far?

**DOI:** 10.1016/j.ebiom.2024.104969

**Published:** 2024-01-18

**Authors:** Su Yin Lim, Helen Rizos

**Affiliations:** a Macquarie Medical School, Faculty of Medicine, Health and Human Sciences, Macquarie University, Australia; b Melanoma Institute Australia, Sydney, Australia

**Keywords:** Immunotherapies, MAPK inhibitors, Dedifferentiation, Cellular states

## Abstract

Over the past decade, there have been remarkable improvements in the treatment and survival rates of melanoma patients. Treatment resistance remains a persistent challenge, however, and is partly attributable to intratumoural heterogeneity. Melanoma cells can transition through a series of phenotypic and transcriptional cell states that vary in invasiveness and treatment responsiveness. The diverse stromal and immune contexture of the tumour microenvironment also contributes to intratumoural heterogeneity and disparities in treatment response in melanoma patients. Recent advances in single-cell sequencing technologies have enabled a more detailed understanding of melanoma heterogeneity and the underlying transcriptional programs that regulate melanoma cell diversity and behaviour. In this review, we examine the concept of intratumoural heterogeneity and the challenges it poses to achieving long-lasting treatment responses. We focus on the significance of next generation single-cell sequencing in advancing our understanding of melanoma diversity and the unique insights gained from single-cell studies.

**Search strategy and selection criteria**

Data for this review were identified by searches of PubMed, and references from relevant articles using the search terms “single-cell RNA sequencing”, “melanoma”, and “treatment response and resistance”. Abstracts and reports from meetings were included only when they related directly to previously published work. Only articles published in English between 1990 and 2023 were included.

## Overview of melanoma

### Melanoma subtypes

Cutaneous melanoma develops on sun-exposed skin and accounts for approximately 95% of all melanoma cases. Cutaneous melanoma is highly metastatic and can spread to local subcutaneous tissues, draining lymph nodes and distant sites throughout the body, with the lung, brain and liver being the most common metastatic sites.^1^ Mucosal and acral melanoma are very rare, accounting for only 1–2% of all melanoma cases in Caucasian populations, although they are more common in Asian countries.^2^ Mucosal melanoma occur on the mucous membranes found in the mouth, nose, throat, anus and genital areas. Conversely, acral melanoma predominantly affects the extremities, including the palms of the hands, soles of the feet and nail beds. Uveal melanoma is another rare melanoma subtype, accounting for 5% of all melanoma cases, and can affect different parts of the uvea including the iris, ciliary body and choroid.^3^

### Management of melanoma

The treatment of cutaneous melanoma has significantly advanced in the past decade due to the introduction of molecular targeted therapies and immunotherapies. Approximately 40–60% of cutaneous melanoma have activating missense mutations in the *BRAF* gene, and another 10–30% of cases have gain-of-function *NRAS* mutations.^4^^,^^5^ These dominant driver mutations induce the activation of the mitogen-activated protein kinase (MAPK) pathway and selective kinase inhibitors of this pathway (e.g., BRAF and MEK inhibitors) have dramatically improved patient outcomes. For instance, 64–70% of patients with advanced BRAF^V600^-mutant melanoma respond to combination BRAF/MEK inhibitors with median overall survival (OS) of 22–34 months compared to the BRAF inhibitor vemurafenib alone (objective response rate (ORR) of up to 50% and median OS of ∼17 months).^6^, ^7^, ^8^ Immunotherapies targeting the immune inhibitory receptors cytotoxic T-lymphocyte antigen 4 (CTLA-4) and programmed cell death 1 (PD-1) enhance T cell-mediated anti-tumour responses. These immune checkpoint inhibitors have similarly improved patient outcomes, with combination anti-CTLA-4/PD-1 generating ORR of 58% and median OS of 72 months (95% confidence interval (CI), 38.2 to not reached) compared to anti-CTLA-4 (ORR of 19% and median OS of 19.9, 95% CI, 16.8–24.6) or anti-PD-1 (ORR of 45% and median OS of 36.9 months, 95% CI, 28.2–58.7) alone.^9^^,^^10^ Although these therapies extend patient survival, most patients will progress on treatment and novel therapies and dosing schedules are underway in the advanced, adjuvant and neoadjuvant settings. The management and treatment of cutaneous melanoma in the primary and metastatic stages have been comprehensively reviewed, most recently by Long et al.^1^

In contrast, less progress has been made in the treatment of acral, mucosal and uveal melanoma (reviewed in^11^) and these melanoma subtypes do not benefit from standard therapies used in the treatment of cutaneous melanoma. For example, compared to cutaneous melanoma, patients with acral or mucosal melanoma have a shorter median OS (17–18 months vs 45 months) when treated with immune checkpoint inhibitors.^12^ Similarly, metastatic uveal melanoma patients treated with immune checkpoint inhibitors show ORR of only 18% and median OS of 19.1 months.^13^ Several promising therapies have recently emerged for uveal melanoma, including combination PKC inhibitor and c-Met inhibitor, which demonstrated an ORR of 45% and a median progression free survival (PFS) of 7 months (https://media.ideayabio.com/). Additionally, a new form of immunotherapy that involves a bispecific CD3 T cell engager that binds gp100 on uveal melanoma cells to initiate an immune-mediated anti-tumour response demonstrated a one-year OS benefit of 73% although this therapy is restricted to uveal melanoma patients with HLA-A^∗^02:01 tumours.^14^

### Treatment resistance in melanoma

The clinical benefit of molecular targeted therapies and immunotherapies is hindered by the development of treatment resistance and the onset of toxicities. Serious toxicities are more commonly observed with combination therapies,^10^ and often necessitate discontinuation of treatment. Durability of responses are also limited with approximately 30% and 50% of melanoma patients progressing after one year of treatment with immune checkpoint or BRAF/MEK inhibitors.^15^^,^^16^ Indeed, the most recent long-term outcomes (6.5 years) of melanoma patients treated with immune checkpoint inhibitors showed median progression free survival (PFS) of 11.5 months for combination anti-CTLA-4/PD-1, 6.9 months for anti-PD-1 and 2.9 months for anti-CTLA-4.^10^

Resistance to BRAF/MEK inhibitors is predominantly due to MAPK reactivation via activation of alternate receptor tyrosine kinases, upstream MAPK regulators and mutations in drug targets. Activation of alternate survival signalling pathways such as the phosphatidylinositiol-3 kinase/protein kinase B (PI3K/AKT) pathways also contribute to BRAF/MEK inhibitor resistance (reviewed in^17^). Mechanisms of immunotherapy resistance can be categorised broadly as tumour-intrinsic or tumour-extrinsic. Tumour intrinsic mechanisms involve alterations in IFNγ, Wnt, MAPK and CDK4/6 signalling, upregulation of immune suppressive molecules and deficiency in antigen presentation while tumour extrinsic mechanisms include immune-poor or immunosuppressive tumour microenvironments devoid of activated effector immune cells due to deficiency in T cell trafficking and infiltration, or T cell exhaustion (reviewed in^18^^,^^19^). Prevalence of these resistance mechanisms appear to be varied; deficiency in IFNγ signalling is uncommon, identified in only 4–10% of progressing tumours,^20^^,^^21^ while we have recently shown that defects in antigen production and presentation are more common, accounting for >70% of progressing tumours analysed.^22^

Importantly, treatment resistance mechanisms are commonly heterogeneous within patients and within tumours, indicating that heterogeneity may be a precursor for poor treatment response and treatment failure.^23^^,^^24^ This is evident in the heterogeneity of tumour responses to PD-1 and BRAF inhibitors,^25^^,^^26^ and likely why predictive markers of immunotherapy response and resistance have large variation and limited specificity and performance.^27^ In keeping with this, no common mechanisms of resistance have been identified in melanoma patients treated with immune checkpoint inhibitors^28^ and interpatient and intrapatient heterogeneity in BRAF/MEK inhibitor resistance have also been identified.^24^^,^^29^ In the following section, we discuss heterogeneity in melanoma tumours, focusing on the source of heterogeneity and the impact on melanoma treatment.

## Intratumoural heterogeneity (ITH) in melanoma

**Fig. 1:**
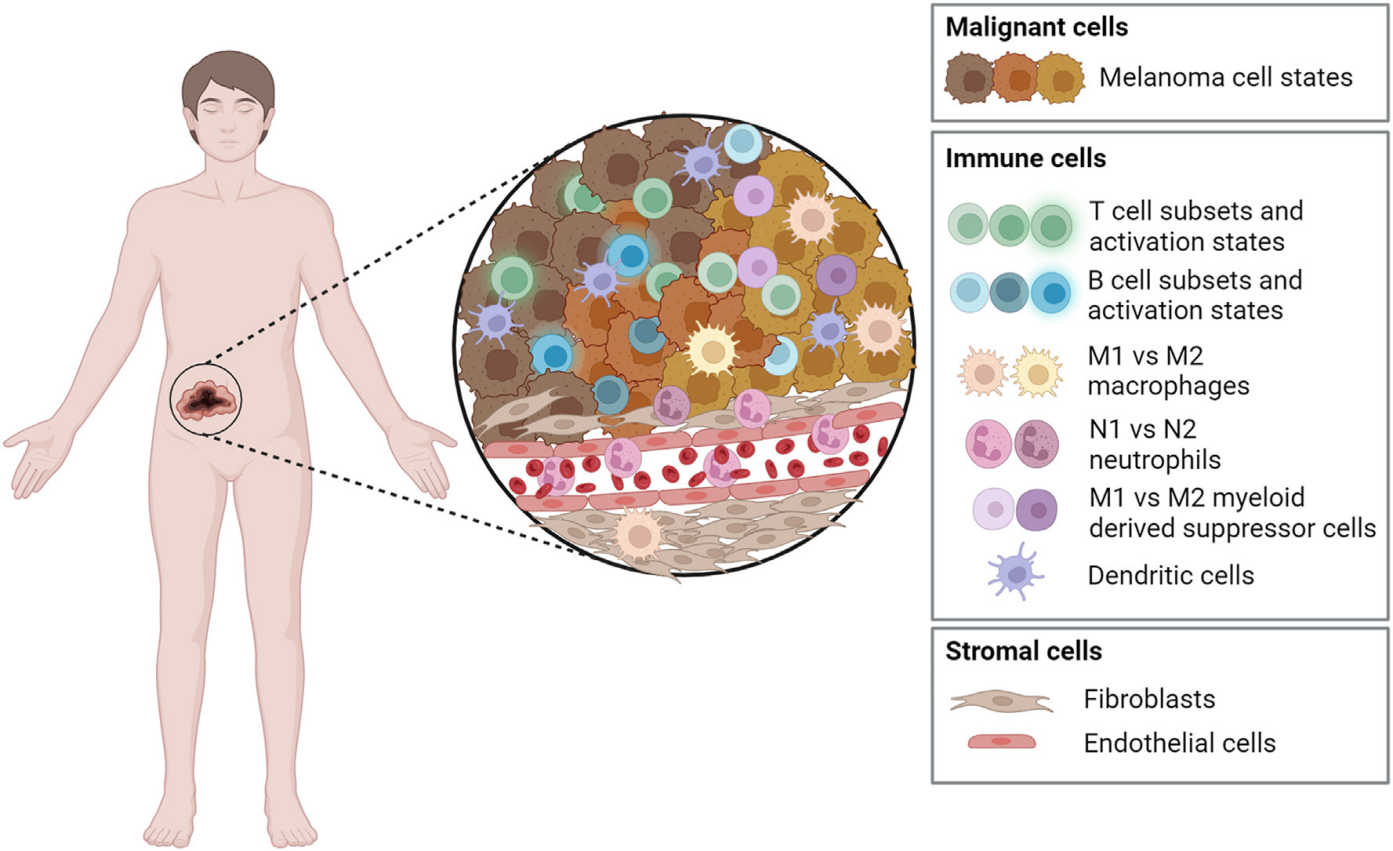
Intratumour heterogeneity is driven by the plasticity of melanoma cells to exist in multiple cells states and the diverse stromal and immune contexture within the tumour.

ITH refers to the distinct molecular and phenotypic profiles of malignant cancer and non-malignant stromal cells within the same tumour specimen. This feature is driven by the plasticity of cancer cells and the presence of heterogeneous stromal cell types in the tumour microenvironment (TME) (Fig. 1).

Molecular differences in cancer cells within a single tumour were initially attributed to genetic diversity, arising from the acquisition of distinct mutations during the evolution of the tumour. However, emerging evidence supports the notion that ITH is largely driven by transcriptional and epigenetic reprogramming of cancer cells in response to environmental cues.^30^ This process, termed phenotypic plasticity, can be reversible and may depend on the type, degree and duration of the stimuli.

### Melanoma plasticity and cellular states

**Fig. 2:**
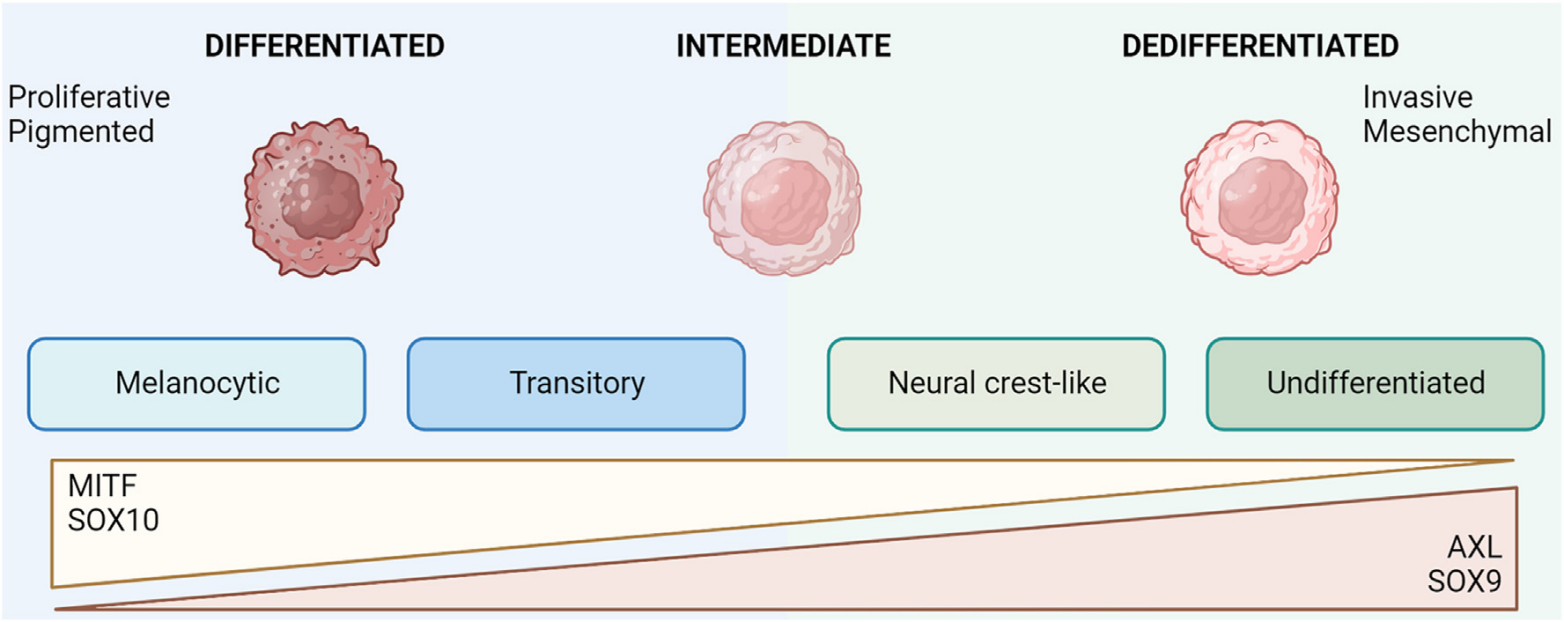
Melanoma cell plasticity and transition to diverse transcriptional and phenotypic states defined by key features.

Melanoma cells are intrinsically plastic and can rapidly adapt and respond to various extracellular stresses, including to drug treatments, inflammation, nutrient and oxygen deprivation.^31^ This adaptation involves a cellular switch to multiple but discrete phenotypes within a single tumour and may be analogous to the epithelial to mesenchymal transition seen in other cancer cell types. However, melanoma cells are not epithelial-like, and instead, the phenotype transition is reversible and occurs across a continuous trajectory, transitioning between a differentiated and a dedifferentiated cellular state.^32^^,^^33^ The differentiated melanoma state is characterised by increased proliferation and expression of pigmentation markers while the dedifferentiated state shows enhanced invasiveness and mesenchymal-like properties; these fundamental features and molecular markers characteristic of the melanoma states are summarised in Fig. 2.

Melanoma phenotypic states were first described in relation to the expression of the melanocyte lineage-specific transcription factor (MITF); differentiated and rapidly proliferating melanoma cells showed high levels of MITF but those with low MITF expression were more invasive and considered dedifferentiated, while an intermediate state displayed variable and moderate MITF expression.^34^ Several intermediate states have since been described based on unique transcriptional signatures or response profiles^33^^,^^35^^,^^36^ (Fig. 2). These cell states are dynamic and can transition in either direction. For instance, when differentiated or dedifferentiated melanoma cells were introduced into immunocompromised mice, the tumours that developed showed mixtures of both cell states, indicating that a particular melanoma state can transition into either phenotype.^32^

### Stromal cell plasticity and phenotypes

Apart from melanoma cells, the differing proportions and types of non-malignant stromal cells in the TME also contributes to ITH. These stromal cell types include immune cells of the myeloid and lymphoid origin and non-immune accessory cells such as fibroblasts with distinct phenotypic and functional diversity (Fig. 1).

TME cells of the myeloid origin include neutrophils, macrophages and myeloid-derived suppressor cells (MDSC). These cells show a high degree of heterogeneity, and similar to melanoma cells, can respond to specific signals by inducing unique transcriptional programs, in a process known as polarisation.^37^ For example, neutrophils, macrophages and MDSCs can be polarised into the classically activated N1 or M1 or the alternatively activated N2 or M2 phenotypes with opposing functions. Classically activated neutrophils, macrophages and MDSCs are pro-inflammatory with anti-tumour activities while alternatively activated myeloid cells are anti-inflammatory and tumour promoting.^38^^,^^39^

Lymphoid T and B cells are also heterogeneous, owing to their ability to differentiate into multiple subsets with discrete functions. T cells are broadly divided into two lineage groups: CD4 and CD8 T cells and can be further subdivided into different subsets including Th1, Th2, Th9, Th17, naïve, effector, memory and regulatory T cells, and ascribed with specific functional states (e.g., dysfunctional, exhausted, activated); these subsets are defined by unique cell surface markers and effector molecules although these markers may be transiently expressed. Each T cell subset also has different antigen specificity, and thus, different T cell receptor (TCR) sequences, all of which influence the functional heterogeneity of T cell responses (reviewed in^40^). Similarly, B cell subsets (e.g., naïve, memory, regulatory, activated) have also been described with distinct functional roles and antigen specificity (reviewed in^41^).

Cancer associated fibroblasts (CAFs) are another important stromal cell type that contributes to ITH. CAFs can undergo phenotypic switching, resulting in differing phenotypes and functional roles.^42^^,^^43^ Altogether, the multiple stromal cell types and phenotypes add further diversity and complexity to ITH.

### Challenges posed to melanoma treatment

ITH is a critical determinant of treatment response and resistance as it provides tumours with added functionality and adaptability. Resistance to treatment and subsequent disease progression and recurrence can develop from the selection and outgrowth of drug resistant cancer cells amongst a heterogeneous tumour population. Due to the drug-induced selection pressure, malignant cells may acquire mutations and/or activate transcriptional and epigenetic programs that confer survival benefits to thrive under pressure.^44^

In cutaneous melanoma, increased ITH has been associated with resistance to molecular targeted therapies and immunotherapies as the plasticity of melanoma cells can differentially influence treatment response. For instance, melanoma cells with the dedifferentiated invasive phenotype are more resistant to BRAF/MEK inhibitor^45^ and appear to display cross resistance to immune-mediated therapies.^46^^,^^47^ Increase ITH also increases the likelihood of melanoma cells to activate diverse and alternate signalling pathways that can circumvent treatment-induced cell death.^48^ Moreover, although increased ITH is accompanied by increased tumour mutational and antigenic load that is more amenable to immunotherapies, a recent study showed that melanoma tumours with high ITH is associated with reduced immune cell infiltration and activation, and decreased overall survival.^49^

The advent of next generation single-cell sequencing technologies has provided a high-level overview and enabled comprehensive analyses to decipher ITH. Studies utilising single-cell analyses have 1) profiled and characterised the complex molecular landscapes of cutaneous, acral and uveal tumours; 2) corroborated presence of distinct melanoma cell states and phenotypes within the same tumour lesion; and 3) functionally dissected the roles of multiple immune cell subsets within the tumours. In the following section, we highlight what these studies have uncovered and what new knowledge they have contributed to our understanding of ITH and mechanisms of melanoma treatment resistance.

## Next-generation single-cell RNA sequencing (sc-RNAseq) in melanoma

### Single-cell RNA sequencing exposed the extent of melanoma plasticity and intratumoural heterogeneity

Several studies have characterised melanoma plasticity in cell culture models using scRNA-seq (Table 1) and these studies illustrate one common finding—that melanoma cells are inherently heterogenous and are able to exist in distinct cell states that differentially adapt to microenvironmental cues. For instance, scRNA-seq was performed on three melanoma cell lines of different genotypes (BRAF-mutant, NRAS-mutant and BRAF/NRAS-wildtype), and while these genotypes formed distinct cell clusters, additional subclusters were identified within each genotype, comprising divergent proliferative, cell cycle, pigmentation or stromal cell gene signatures.^50^^,^^51^ Distinct cell clusters were also identified from the scRNA-seq of 10 melanoma cell lines, demonstrating the diversity between melanoma cultures.^36^ Based on the differential expression of transcription factors involved in melanoma phenotype switching (e.g., SOX9 and SOX10), these clusters were further categorised as melanocytic, intermediate or mesenchymal-like. Phenotypically, these states differed in their migratory capabilities, and although these states are controlled by conserved and distinct gene regulatory networks and appear to be stable in culture over time,^36^ they can be easily perturbed by exogenous stimulation. For example, scRNA-seq of BRAF-mutant melanoma cells treated with the BRAF inhibitor vemurafenib, alone or in combination with MEK inhibitor cobimetinib or trametinib, showed seven separate cell clusters representative of distinct transcriptional states. Each treatment group showed enrichment of particular transcriptional states,^52^ suggesting that the frequencies of these states change with treatment, and this dynamic change contributes to the overall heterogeneity. Similarly, in NRAS-mutant melanoma cells treated with combination MEK and CDK4/6 inhibitors, scRNA-seq revealed distinct cell clusters reflecting heterogeneity in transcriptional response to treatment, and differences in response was postulated to cause outgrowth of intrinsically resistant melanoma subpopulations.^53^

**Table 1:** Single-cell RNA sequencing studies of melanoma cell cultures.

Study sample	Cell types studied	Key findings	Data source
Three melanoma cell cultures	Melanoma cells	Characterised three distinct melanoma cell states: proliferative, pigmentation and stromal, and the bipolar MIFT/AXL expression in these cells. These findings suggest MITF and AXL as potential markers to identify specific stages of tumour progression.	GSE81383^50^^,^^51^
Ten melanoma cell cultures	Melanoma cells	Identified shared gene regulatory networks that mediate transition of melanocytic and mesenchymal cell states. This study proposed the existence of an intermediate state during phenotype switching between the melanocytic and mesenchymal states.	GSE134432^36^
One BRAF-mutant melanoma cell line with 4 treatment conditions	Melanoma cells	Identified cell trajectories associated with resistance to single and combination MAPK inhibitors. This study proposed therapeutic targets to circumvent treatment resistance.	GSE164897^52^
Four NRAS-mutant melanoma cell lines treated with MEK and CDK4/6 inhibitors	Melanoma cells	Differentiated distinct early vs late transcriptional profiles in response to drug treatment. This study identified the ATP-gated ion channel P2RX7 as a potential target in delaying acquired resistance to CDK4/6 inhibitors.	GSE230538^53^

Plasticity and heterogeneity of melanoma cells have also been validated in melanoma tumours. scRNA-seq of melanoma cells isolated from patient-derived xenograft mouse models treated with BRAF/MEK inhibitors identified four transcriptional states with differential MITF activity representative of the pigmented, invasive, neural crest stem cell and starved phenotypes. These cell states showed dynamic transcriptional trajectories in response to drug exposure, transitioning from a proliferative to a starved state, followed by a pigmented to a neural crest stem cell state.^33^

In addition to characterising melanoma plasticity, scRNA-seq has also been utilised to dissect ITH (Table 2). For instance, scRNA-seq of one primary acral melanoma and 18 cutaneous melanoma tumour biopsies, including melanoma metastasis to lymphoid tissues and distant organs, showed heterogeneity in both the melanoma and non-malignant cell populations.^54^ Melanoma cells differed in their expression of cell cycle genes with two divergent groups: high-cycling vs low-cycling, and in their expression of MITF: MITF-high vs MITF low. Diversity of T cells in these tumours was also reported, with the identification of different T cell subsets (e.g. CD4, CD8, T regulatory) and activation states,^54^ and these findings were corroborated in an expanded cohort of cutaneous melanoma patients^55^ and in scRNA-seq studies of isolated immune cells from cutaneous melanoma tumours.^56^, ^57^, ^58^ Importantly, these subsequent studies have provided in-depth characterisation of the diverse transcriptional states of CD8 and NK cells that reflect their phenotypic and functional differences.

**Table 2:** Single-cell RNA sequencing studies of melanoma tumours.

Study sample	Cell types studied	Key findings	Data source
Tumours from patient-derived xenograft models treated with BRAF/MEK inhibitors	Melanoma cells	Identified transcriptional cell states, including the neural crest stem cell transcriptional program, associated with drug response and tolerance. These findings highlight neural crest stem cells to be key drivers of treatment resistance.	GSE116237^33^
Cutaneous melanoma tumours (n = 18) metastatic to lymph nodes and distant sites, and primary acral melanoma (n = 1).	Melanoma and immune cells	Characterised melanoma heterogeneity in cell cycle and MITF expression, and distinct T cell functional states. These findings highlight the transcriptional heterogeneity that exists within the same melanoma tumour.	GSE72056^54^
Cutaneous melanoma tumours (n = 33); untreated, resistant or responsive to immune checkpoint inhibitors	Melanoma cells	Identified a melanoma-intrinsic program associated with T cell exclusion and immunotherapy resistance. These findings indicate innate treatment resistance to be driven by a transcriptional program.	GSE115978^55^
Cutaneous melanoma tumours (n = 48) treated with immune checkpoint inhibitors; immune cells were isolated and analysed	Immune cells	Characterised two distinct CD8 T cell transcriptional states associated with immunotherapy response and resistance. These findings identify the TCF7 transcription factor to predict immunotherapy response.	GSE120575^56^
Cutaneous melanoma tumours (n = 29), untreated or treated with immune checkpoint inhibitors; immune cells were isolated and analysed	Immune cells	Characterised CD8 T cell transcriptional states associated with effector function and dysfunction. These findings indicate that the CD8 T cell dysfunction signature is associated with tumour reactivity.	GSE123139^57^
Cutaneous melanoma tumours (n = 5) treated with immune checkpoint inhibitors; immune cells were isolated and analysed	Immune cells	Characterised NK cell transcriptional states associated with specialised functions. These findings highlight the distinct functional phenotypes of NK cells in tumours.	GSE139249^58^
Treatment-naïve melanoma brain metastases (n = 22) and extracranial melanoma metastases (n = 10)	Melanoma and immune cells	Characterised genomic and transcriptional heterogeneity in intracranial and extracranial melanoma brain metastases. These findings indicate that intracranial melanoma brain metastases are more chromosomally unstable and adopt a neuronal-like cell state.	GSE185386^59^
Primary (n = 8) and metastatic (n = 3) uveal melanoma tumours	Melanoma and immune cells	Characterised uveal melanoma transcriptional heterogeneity and T cell diversity. These findings identify uveal melanoma tumours to be infiltrated by LAG3-expressing CD8 T cells.	GSE139829^60^
Primary uveal melanoma tumours (n = 6)	Malignant cells	Characterised distinct transcriptional cell states within uveal tumours. These findings identify a gene regulatory network underlying the invasive state driven by the transcription factor HES6.	GSE138433^61^
Primary uveal melanoma tumours (n = 6)	Malignant cells	Identified uveal melanoma progression driven by loss of Polycomb repressive complex 1. These findings suggest that deregulation of the complex can promote tumour progression.	GSE160883^62^
Acral (n = 5) and cutaneous (n = 3) melanoma tumours	Melanoma and immune cells	Characterised acral melanoma and immune cell heterogeneity. These findings indicate that acral melanoma tumours are dominated by immunosuppressive states compared to cutaneous melanoma.	GSE215121^63^
Primary (n = 5) and metastatic (n = 4) acral melanoma tumours	Melanoma and immune cells	Charaterised acral melanoma and immune cell heterogeneity. These findings further confirm that acral melanoma tumours have more suppressive immune environments compared to cutaneous melanoma.	GSE189889^64^
Primary (n = 4), adjacent (n = 3) and metastatic (n = 1) acral melanoma tumours	Melanoma and immune cells	Characterised acral melanoma and immune cell heterogeneity. These findings identify key genes such as TWIST, EREG and TNFRSF9 that could drive the deregulation of the tumour microenvironment.	HRA001804^65^

scRNA-seq of cutaneous melanoma metastatic to the brain and extracranial sites also supported ITH, with multiple malignant and non-malignant cell types and states identified. Both intracranial and extracranial brain metastases showed heterogeneity in melanoma cells, which primarily differed in their cell cycling status. Intracranial and extracranial metastases were also transcriptional distinct with intracranial metastases highly represented by melanoma cells with neuronal-like features. Similarly, distinct differences were noted in the diversity of the non-malignant immune cell populations, with more dysfunctional CD8 T cells and myeloid cells in intracranial metastases.^59^

ITH has been similarly reported in other melanoma subtypes using scRNA-seq, including in uveal and acral melanoma. For example, scRNA-seq analysis of primary and metastatic uveal melanoma tumours identified four tumour cell clusters (BAP1 mutant vs BAP1 wildtype; PRAME negative vs PRAME positive) with 16 discrete transcriptional cell states, and multiple T cell subsets characterised by activation and exhaustion markers^60^; the latter was surprising given that uveal melanoma is typically considered to be immune cell-poor. Presence of discrete uveal melanoma transcriptional cell states were further supported by several scRNA-seq studies of primary uveal melanoma tumours.^61^^,^^62^ These studies additionally showed genomic and phenotypic diversity of uveal melanoma cells, but reported limited infiltration of immune cells, likely due to inherent site-specific differences in primary vs metastatic uveal tumours.

In acral melanoma tumours, scRNA-seq identified five melanoma cell clusters differing in their expression of oncogenic signalling pathways (e.g., Wnt, TGF-β, Type I IFN) gene sets. Diverse immune cell subsets were also identified, including CD4 and CD8 T cells with multiple functional states although the immunosuppressive state (e.g., exhausted CD8 T cells with high PD1 and TIM-3 expression) was predominant.^63^ Melanoma and immune cell diversity in acral melanoma tumours were recapitulated in two other scRNA-seq studies. The study by He et al. reported eight melanoma clusters, separated by their stages of differentiation and differential expression of epithelial-to-mesenchymal markers and response to TGF-β.^65^ Even more distinct transcriptional cell states were identified by Li et al. and these 16 acral melanoma states differed in their enrichment of genes involved in various biological processes (e.g., telomere maintenance, cytoskeletal remodelling, immune response) and oncogenic signalling (e.g., Wnt, IFN).^64^ The 16 acral melanoma cell states were associated with previously reported signatures of melanoma dedifferentiation,^35^^,^^54^ highlighting the plasticity of acral melanoma cells to transition between differentiation phenotypes similar to cutaneous melanoma. It is also interesting to note that all three scRNA-seq studies on acral melanoma tumours showed considerable lymphocyte heterogeneity, with presence of diverse B and T cell functional states,^65^ although when compared to cutaneous melanoma, acral melanoma tumours had less immune cell infiltration^64^ and were dominated by immunosuppressive T regulatory cells and exhausted CD8 T cells.^63^

Overall, these scRNA-seq studies established that ITH is driven by the plasticity of melanoma cells to exist and transition between different cellular states and the diversity of multiple immune cell types and functional states in the TME. Of note, scRNA-seq analyses enabled discrete melanoma cell populations to be further defined by their differential gene expression and regulatory networks, and for the functional status of immune cell populations to be inferred by their transcriptional signatures, and these would not be possible with traditional bulk RNA sequencing. Although scRNA-seq studies have advanced our understanding of the biology and the complexity of melanoma tumours, it is important that we translate these findings to inform on treatment response and resistance, as discussed in the next section.

### Single-cell RNA sequencing elucidated mechanisms of tumour progression, treatment response and resistance

scRNA-seq has the potential to comprehensively dissect the signalling networks that govern tumour progression, treatment response and resistance, and thus improve and inform new therapeutic approaches. For instance, scRNA-seq studies of melanoma cultures exposed to different treatments showed induction of distinct transcriptional programs that could lead to drug resistance; BRAF-mutant melanoma cells resistant to BRAF inhibitor alone showed a high cell cycling and proliferative phenotype while those resistant to combination BRAF/MEK inhibitors showed signatures of high translational activity, PI3K pathway activation and alternate MAPK pathway reactivation.^52^ Treatment of NRAS-mutant melanoma cells with MEK and CDK4/6 inhibitors induced two divergent drug responses: fast-adapting cells characterised by increased ion transmembrane transport and an immune-like state, and slow-adapting cells with features of senescence.^53^ Hence, it is provocative to suggest that understanding the mechanisms of treatment response may provide novel approaches to circumvent melanoma cell adaptation and prevent the emergence of drug resistance.

Resistance to immunotherapy has been described as innate or primary resistance (the cancer does not respond to treatment from the onset), acquired resistance (the cancer initially responds to treatment, but will eventually relapse or progress after a period of time) or adaptive resistance (the cancer is recognised by the immune system but adapts to the inflammatory immune response to evade immune attack).^66^ The specific mechanisms of treatment resistance may provide a rational approach to restore treatment response. For instance, Rambow et al. demonstrated the emergence of four distinct drug-tolerant states that arise during treatment using scRNA-seq.^33^ In particular, melanoma cells with neural crest stem cell state were enriched after treatment with BRAF/MEK inhibitors, and this cell state transition was mediated by retinoid X receptor signalling. Targeting this signalling pathway delayed tumour progression, suggesting that neural crest stem cell melanomas are resistant to BRAF/MEK inhibitors and thus, may drive tumour recurrence. An innate CDK4/6-driven transcriptional program that reduced the physical interactions between melanoma cells and T cells, leading to the exclusion of T cells in melanoma tumours was also identified using scRNA-seq analysis.^55^ Importantly, CDK4/6 inhibition repressed this T cell exclusion program and enhanced immunotherapy response in pre-clinical melanoma models.^55^ Altogether, scRNA-seq studies provide invaluable insights into treatment resistance, tracing the dynamic evolution of resistance at the single cell level and providing novel mechanistic-based approaches to circumvent drug resistance.

In addition to uncovering tumour-intrinsic treatment resistance mechanism, several scRNA-seq studies have leveraged the single-cell data to provide details of tumour-extrinsic mechanisms of response and resistance. For example, Sade-Feldman et al. identified two distinct CD8 T cell states in melanoma tumours treated with immune checkpoint inhibitors: CD8_G state enriched for genes involved in memory, activation and cell survival vs CD8_B state enriched for genes associated with cell exhaustion.^56^ The CD8_G state was characterised by TCF7 expression, and infiltration of TCF7^+^ CD8 T cells was associated with better tumour regression. However, the exhausted CD8_B state or the presence of exhausted CD8 T cells does not necessarily predict immunotherapy resistance, as exhausted or dysfunctional CD8 T cells show transcriptomic signatures of proliferation and differentiation, and high clonal expansion indicative of tumour reactivity.^57^

Taken together, scRNA-seq data can be applied to better understand how distinct melanoma cell states differentially adapt and acquire resistance to treatment, and to dissect the roles of discrete immune cell subsets in treatment response. These intricate analyses are not achievable with bulk RNA sequencing. Importantly, scRNA-seq datasets are publicly available and the transcriptome data can be further mined and utilised for meta-analysis. It is worth noting that this review focuses on initial scRNA-seq studies (Tables 1 and 2) and these studies have also propelled the development and validation of new methodologies and tools for scRNA-seq analysis, including cell type classification,^67^ trajectory inference,^68^ analysis of cell–cell communication^69^ and interactions among genes.^70^

### Towards single-cell spatial transcriptomics and multiomics

In addition to understanding the molecular and transcriptomic changes at the single-cell level, there is now increasing interest in mapping the spatial distribution of individual cells. Single-cell spatial transcriptomics offers the added advantage of examining the interplay and local intercellular communication between different cell types in the TME and provides tissue context to understand cell interactions and localisation within a preserved tissue architecture. For example, spatial transcriptomics performed on seven intracranial and four extracranial melanoma brain metastases identified distinct clusters of lymphoid aggregates and spatially restricted expression of MHC I antigen presentation and IFN response genes amongst cancer cells, suggesting that distinct regions within the tumour may be more susceptible to treatment based on the cellular and transcriptional differences.^59^

scRNA-seq can be additionally integrated with other next generation sequencing and omics approaches to provide more information and granularity of the single cells analysed, and to better characterise the diverse melanoma and immune cell states and phenotypes. Single cell multiomics technologies now allow the simultaneous profiling of cell surface protein expression,^71^ the genome, the DNA methylome and the epigenome based on chromatin accessibility (reviewed in^72^). In melanoma, these integrated technologies have further contributed to a better understanding of melanoma biology and treatment response and resistance. For instance, to identify immunotherapy resistance mechanisms, a CRISPR-Cas9-mediated perturbation model involving patient derived melanoma cells and matched tumour infiltrating lymphocytes was combined with scRNA-seq and epitope sequencing (collectively called Perturb-CITE-seq) to profile the single-cell transcriptome and expression of 20 surface proteins in a CRISPR/Cas9 loss-of-function screen. Using this approach, loss of CD58 was shown to confer immune evasion to T and NK cell-mediated killing.^73^

## Exploiting single cell technologies—where do we go from here?

scRNA-seq is expensive and can amount to 20 times that of traditional bulk RNA sequencing. Moreover, bulk RNA sequencing only requires up to 20 million sequencing reads per sample while the number of reads per cell for scRNA-seq may range from 50,000 to 150,000 depending on the biological question. In a sample where 5000 targets cells are captured, this equates to 750 million sequencing reads per sample, thus adding to the experimental cost. The analysis of rare cell subsets is also challenging, and these cells may need to be enriched during single cell preparation or the sequencing depth needs to be increased. scRNA-seq also requires specialised kits and instruments for single cell partitioning and barcoding, and these are costly and may not be widely accessible. Furthermore, scRNA-seq data are inherently more variable and complex than bulk RNA data, and necessitate intricate and sophisticated computational pipelines to ensure quality control, maintain data integrity, accuracy and reproducibility.

To date, there have been more than 200 research articles (PubMed search using the algorithm “single-cell RNA sequencing” AND “melanoma”) and yet the translational and fundamental value of these data compared to bulk RNA sequencing remains unclear. Bulk RNA sequencing remains more routine, affordable and accessible, and will provide adequate information on the transcriptional changes occurring within a tumour sample. Certainly, the most valuable information provided by scRNA-seq studies is the characterisation of ITH, including the more precise identification of diverse melanoma and immune cell subsets and states within a single tumour lesion. Bulk RNA sequencing will only provide an average expression of signals and even though several methods have been developed to deconvolute bulk RNA data to provide information on distinct cell populations using reference gene signatures (e.g. CIBESORTx, MuSiC),^72^^,^^74^ these methods are not precise and bulk analyses are limited in discerning the complex cellular content of tumours. scRNA-seq studies have also captured the transient and poorly represented cellular and transcriptional states that are typically masked by bulk RNA sequencing. This is particularly relevant as drug treatment induces melanoma cells to adopt diverse transcriptional response profiles and drives the generation of resistant cell states that are molecularly, morphologically and functionally distinct.^75^ These drug adaptations change fluidly with time and may be different between distinct cell states, and these changes can only be detected by scRNA-seq but not by bulk RNA sequencing.

## Outstanding questions

The characterisation of the diversity of functional states in melanoma and immune cells not only supplemented our comprehension of how melanoma tumours evolve and respond to treatment, but also provided potential therapeutic targets to circumvent treatment resistance. For example, identifying effectors that regulate melanoma cell plasticity or mechanisms underlying melanoma cell state transitions may present opportunities to reverse or prevent the emergence of treatment resistant cell states. Moreover, the ability to pinpoint the onset of these transitions can allow us to repurpose current therapies or utilise multiple combination therapies to target the diverse cell states, thus eliminating resistant melanoma clones that may persist and cause tumour recurrence. Future research should consider ITH and the existence of these diverse melanoma cell states and this may only be possible by implementing scRNA-seq to 1) dissect the presence and abundance of distinct melanoma cellular states, 2) uncover how each state respond to treatment, 3) identify mediators regulating the cell state transition, and 4) elucidate the timing and trajectory of how these states emerge. Thus far, bulk transcriptome analysis has not led to the identification of therapeutic targets or approaches of overcoming treatment resistance that have translated to the clinic. It remains to be seen if single cell transcriptomics can hold such promise.

## Contributors

SYL and HR conceptualized, wrote, reviewed and edited the manuscript. All authors have read and approved the final version of the manuscript.

## Declaration of interests

All authors declare that they have no competing interests.
